# Benzothiadiazole oligoene fatty acids: fluorescent dyes with large Stokes shifts

**DOI:** 10.3762/bjoc.12.270

**Published:** 2016-12-14

**Authors:** Lukas J Patalag, Daniel B Werz

**Affiliations:** 1 Institut für Organische Chemie, Technische Universität Braunschweig, Hagenring 30, 38106 Braunschweig, Germany

**Keywords:** fatty acid, fluorescence, lipid, membrane, Stokes shift

## Abstract

Herein, we report on the synthesis and characterization of novel fluorescent fatty acids with large Stokes shifts. Three examples consisting of the same number of carbon atoms and thus of similar chain length are presented differing in their degree of unsaturation. As major fluorogenic contributor at the terminus benzo[*c*][1,2,5]thiadiazole was used. Respective syntheses based on Wittig reactions followed by iodine-mediated isomerization are presented. The absorption properties are modulated by the number of conjugated C=C double bonds of the oligoene chain ranging from one to three. Large Stokes shifts of about 4900–5700 cm^−1^ and fluorescence quantum yields of up to 0.44 were observed.

## Introduction

**Figure 1:**
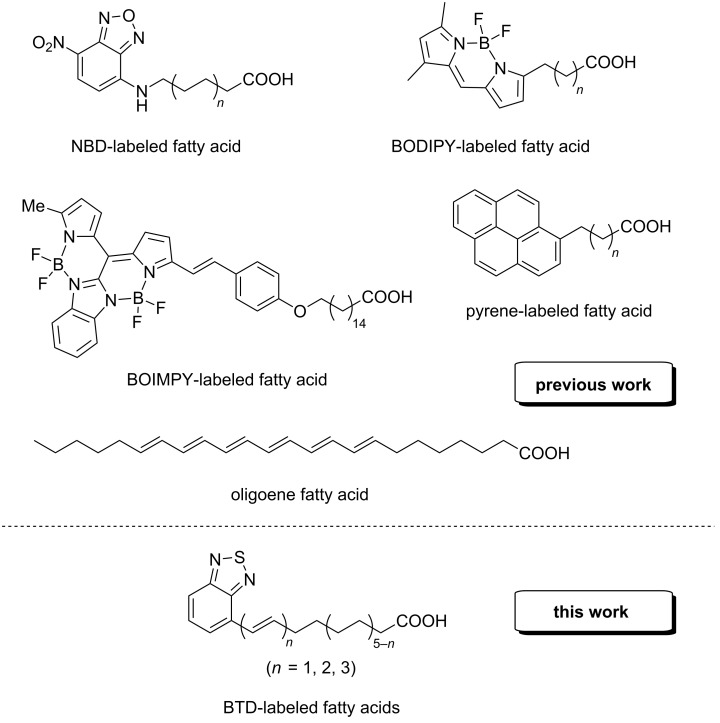
Examples for previously prepared fluorescent fatty acids and our present work.

The membrane of living cells consists of a variety of lipids. More than 40 years ago, biological membranes were first described as Fluid Mosaic in which proteins were embedded [1]. During recent decades it became more and more clear that such a simple model is not sufficient to understand membrane dynamics and function. Often membrane domains are formed in which certain lipids, glycolipids or proteins are enriched [2–4]. Such domains – also called lipid rafts – do not only differ in their chemical composition, but also show different physical properties (e.g., differences in membrane thickness and stiffness, different diffusion coefficients etc.) [5–6]. Tools to investigate lipid membranes are multifaceted; however, all optical methods are hampered by the missing absorption and fluorescence properties of natural occurring lipid components. Therefore, indirect methods are commonly employed. Either unnatural fluorescent dyes are inserted into the membrane (e.g., pyrene) or the hydrophilic part of lipids is utilized for the covalent attachment of fluorophores. Another possibility is the use of fluorescently labelled antibodies which bind membrane components such as the carbohydrate part of glycolipids [7–8]. A further alternative is to render the lipid and especially the fatty acid part fluorescently active by the introduction of fluorescent moieties (Figure 1). Prominent examples in this area are NBD- (**n**itro**b**enzoxa**d**iazole) [9–10], BODIPY- (**bo**ron-**dipy**rromethene) [11–12], BOIMPY- (bis(**bo**rondifluoride)-8-**im**idazodi**py**rromethene) [13] and pyrene-labeled fatty acids [14]. Of course, all these alterations might also affect the membrane structure and its dynamics. While the NBD-fluorophore suffers from unsuitable polarity, a pyrene motif disrupts the unpolar membrane core with high bulkiness. BODIPY and BOIMPY scaffolds on the other hand expose fluoride residues which might be able to interact with polarized H–X bonds. Therefore, we synthesized pentaene and hexaene fatty acids which bear five or six double bonds at the terminus or in the middle of the acyl chain [15]. Their slim shape mimics the natural geometry of a saturated hydrocarbon chain and should therefore only lead to minimal disturbances [16]. Nevertheless, we found that their stability with respect to both, oxygen and strong laser beams, is relatively low. The design of novel fluorescent fatty acids is therefore a challenging tightrope walk between advantageous spectroscopic properties, overall stability and a non-interfering molecular shape. As a promising contribution we designed alternative fatty acids which are constructed as a combination of double bonds and benzo[*c*][1,2,5]thiadiazole as a relatively unpolar terminal headgroup (Figure 1). Its electron-withdrawing strength adds on the one hand significant stability towards acidic environments and should furthermore trigger a red-shift in absorption. As another strategic goal the fluorescent fatty acids were supposed to be equipped with very similar geometrical parameters differing only in their absorption and emission wavelengths. The grade of unsaturation as the sole geometrical difference thus provides a set of probes to study the effect of rigidified ethene moieties as straight-chain alkane surrogates within biological membranes.

## Results and Discussion

### Synthesis

The most prominent methods to access oligoene structures are either cross-coupling reactions [17–19] or Wittig-type reactions [20–22]. The advantage of the latter ones is that they are often conducted at low temperatures and therefore are employed for sensitive compounds. However, a drawback of Wittig reactions is the fact that the stereochemistry of the emerging double bond strongly depends on the type of substituent used. Aliphatic residues tend to give the (*Z*)-isomer. If the thermodynamically more stable (*E*)-isomer is needed, a subsequent isomerization has to take place.

**Scheme 1:**
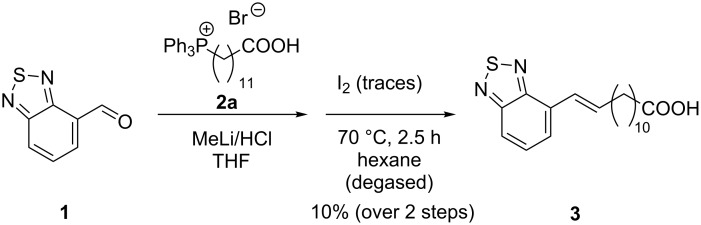
Synthesis of fatty acid **3** with one olefinic unit.

To access the benzothiadiazole (BTD) fatty acid **3** with just one conjugated double bond we made use of the Wittig reaction starting with commercially available aldehyde **1**. As expected, the (*Z*)-isomer was the major product; thus, we performed a subsequent *cis–trans* isomerization with traces of iodine as catalyst (Scheme 1). It proved to be crucial to employ degassed hexane and to ensure a strict exclusion of oxygen. Considering both, the isomerization was finished just by removing the solvent while the yield of compound **3** was not hampered.

**Scheme 2:**
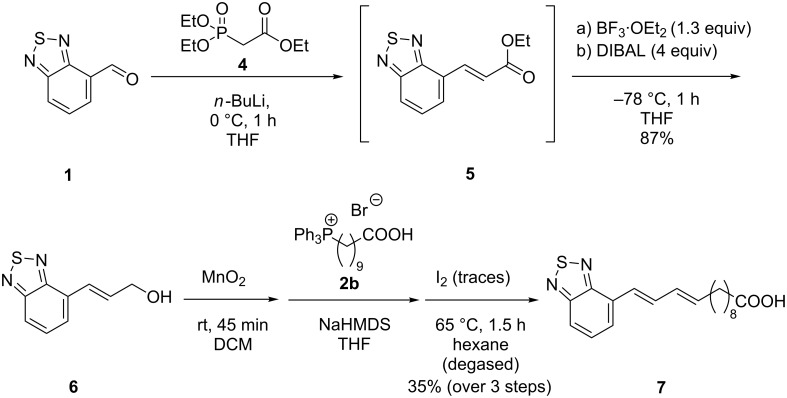
Synthesis of fatty acid **7** with two olefinic units.

For a BTD fatty acid analogue of the same length, but of more extended conjugation we made use of the Horner–Wadsworth–Emmons (HWE) reaction. Phosphonate **4** was reacted with the respective aldehyde **1**. In a facile three-step one-pot process the emerging α,β-unsaturated ester **5** was immediately converted to the alcohol **6** in 87% yield in the presence of a Lewis acid and DIBAL at low temperatures. MnO_2_-mediated oxidation afforded the respective aldehyde that was immediately transformed by Wittig reaction. Iodine-catalyzed *cis–trans* isomerization yielded the desired fatty acid **7** in 35% over three steps (Scheme 2).

**Scheme 3:**
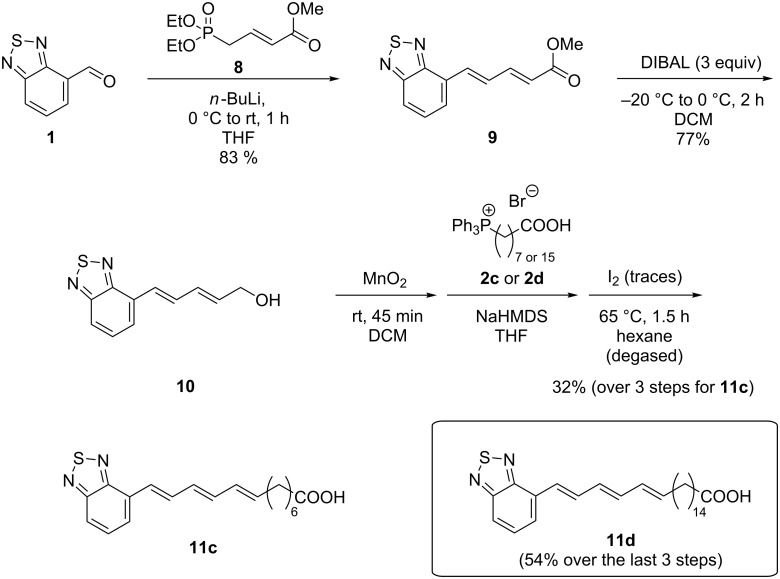
Synthesis of fatty acid **11c** with three olefinic units.

The analogue with three conjugated double bonds was accessed by a similar route that differs only in the type of the phosphonate being employed as starting material. Since three double bonds are required a tailor-made α,β-unsaturated phosphonate **8** was used, which we already employed successfully in former oligoene syntheses [15]. Whereas the second double bond is the result of the HWE reaction the third one is generated in the final Wittig reaction furnishing BTD-equipped triene fatty acid **11c** in an overall yield of 32% (Scheme 3). Notably, the ester intermediate **9** showed already a strong fluorescence at ambient light. By choosing a Wittig salt with a longer alkyl chain the synthesis of the fatty acid **11d** with a chain of 21 carbon atoms in addition to the BTD unit was equally feasible.

### Spectroscopic properties and quantum chemical calculations

**Figure 2:**
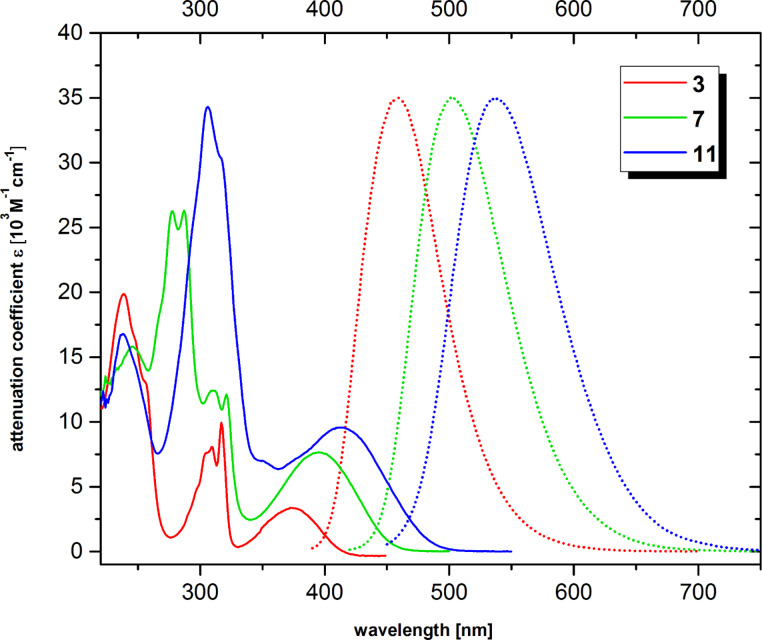
Absorption spectra of fatty acids **3**, **7** and **11**. Solid lines show the UV absorption while dashed lines show fluorescence emission. Excitation was performed at the longest wavelength in each case.

If one follows the oligoene chain and includes the two s-*cis*-shaped double bonds of the BTD headgroup one could regard the fluorogenic core as an oligoene with a geminal diimine acceptor group. Since oligoene absorptions are well-known we anticipated here to access a somehow red-shifted level of excitation energy. Indeed, a bathochromic shift of more than 60 nm relative to a corresponding underivatized oligoene moiety was observed in each case. As anticipated, λ_max_ values increase with a growing number of double bonds from **3** via **7** to **11** (Figure 2).

Concomitantly, the rather low attenuation coefficient at the longest wavelength absorption rises from 3.400 M^−1^cm^−1^ (**7**) to 11.000 M^−1^cm^−1^ for fatty acid **11** with three conjugated double bonds (Table 1). Fluorescence excitation spectra (not shown) for all three fluorophores pretty much coincide with the corresponding absorption spectra in Figure 2, which allows the fluorescence to be switched on at the longest wavelengths respectively.

**Table 1:** Spectroscopic data of synthesized fatty acids in THF at room temperature. Attenuation coefficients ε refer to the longest wavelength absorption peaks respectively.

	λ^A^_max_ [nm]	λ^F^_max_ [nm]	ε [10^3^ M^−1^cm^−1^]	Δ 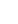 [cm^−1^]	φ_F_ (rt)	Brightness [10^3^ M^−1^cm^−1^]

**3**	374	459	3.4	4950	0.44	1.5
**7**	395	501	7.6	5360	0.33	2.5
**11c**	412	537	9.6	5650	0.18	1.7
**11d**	412	537	11.0	5650	0.18	2.0

**Figure 3:**
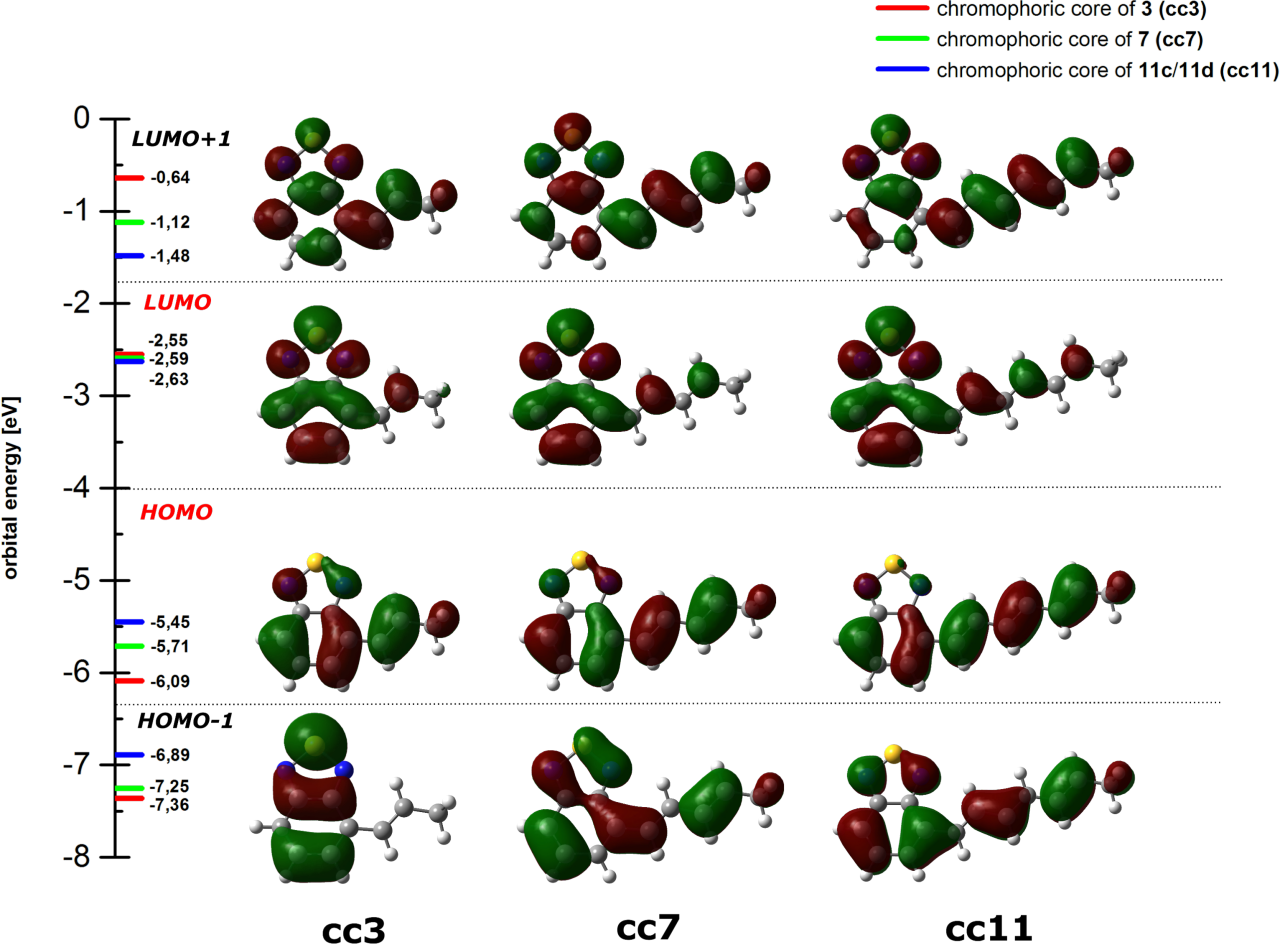
Frontier orbital energies (DFT) and their pictorial representation for the chromophoric cores (cc) of **3**, **7**, and **11**.

Stokes shifts are large (≈5000 cm^−1^) and increase in the same manner with the size of the π-system albeit to a smaller extent. These findings can be quite consistently rationalized when the frontier orbitals of the chromophoric cores (cc3, cc7, cc11) are taken into account. DFT calculations (B3LYP/6-311G(d,p)) reveal that the HOMO is predominantly located at the oligoene chain while the LUMO is rather spread over the BTD terminus (Figure 3).

Indeed, further TD-DFT calculations (not shown) assign the longest wavelength absorptions of all three compounds to a high contribution of a HOMO–LUMO transition. Thus, the low overlap between both wavefunctions results in a small transition dipole moment and subsequently a quite weak absorption. The situation is even aggravated by the specific small angle (≈64°) between the oligoene and the longitudinal axis of the BTD moiety, which diminishes the dipole moment of the electronic shift additionally. However, the large displacement of electron density from one part of the π-system to another enforces a geometrical equilibration of the nuclei which triggers the emission event to take place from a considerably relaxed excited state. As a consequence, the fluorescence is significantly red-shifted, Stokes shifts are large and fluorescence efficiencies decrease when the expansion of the oligoene moiety induces a pronounced geometrical relaxation upon excitation. Furthermore, as double bonds are added, the structure starts to resemble polyene systems whose fluorescence quantum yields are known to be quite modest. For comparison, a prototypical pentaene motif exhibits a fluorescence quantum yield of 0.06 in EtOH [23]. Our fatty acids **11c** and **11d**, however, which can be regarded as terminally substituted pentaene derivatives, can still reach values of 0.18 in THF.

## Conclusion

In conclusion, we have developed a robust approach to three oligoene-shaped fluorescent fatty acids whose fluorescence efficiency is markedly boosted by an unpolar benzo[*c*][1,2,5]thiadiazole (BTD) moiety as terminal headgroup. Wittig reactions in combination with Horner–Wadsworth–Emmons reactions proved to be the method of choice to access the desired structures, which vary only in the grade of unsaturation while the length of their hydrocarbon chain is maintained. The absorption properties were thus modulated by the number of double bonds ranging from λ^A^_max_ = 374 nm (one double bond) to λ^A^_max_ = 412 nm (three double bonds) which matches the requirements for modern laser equipments to trigger efficient excitation. All three variants show remarkably large Stokes shifts ranging from 4900–5700 cm^−1^ and fluorescence quantum yields in the range of 0.18 to 0.44. As a set of geometrically similar, but spectroscopically different fluorescent probes we believe that these fatty acids might find interest as useful candidates to study the sensitive hydrophobic area of membranes in terms of domain formation or as labeling agents in general.

## Experimental

**General.** All solvents for column chromatography were distilled before use unless otherwise stated. Tetrahydrofuran (THF) and diethyl ether (Et_2_O) were distilled from sodium/benzophenone under an argon atmosphere. Dichloromethane (CH_2_Cl_2_) and tetrachloromethane (CCl_4_) were distilled from CaCl_2_ under an argon atmosphere. All other solvents were used as analytical grade and were stored over suitable molecular sieves (3 Å or 4 Å). Air and moisture sensitive reactions were carried out in oven-dried or flame-dried glassware, septum-capped under atmospheric pressure of argon. Commercially available compounds were used without further purification unless otherwise stated. Absolute fluorescence quantum yields were determined using a PTI QuantaMaster 40 UV–vis spectrofluorometer equipped with an integrating sphere. The provided corrections for excitation and emission were applied.

**(*E*)-13-(Benzo[*c*][1,2,5]thiadiazol-4-yl)tridec-12-enoic acid (3):** The corresponding Wittig salt (165 mg, 0.305 mmol, 1.0 equiv) was suspended in THF (1 mL). Methyllithium (1.5 M in diethyl ether, 0.44 mL, 0.670 mmol, 2.2 equiv) was added at −78 °C and the reaction mixture was warmed to rt over 30 min. A solution of aldehyde **1** in THF (1 mL) was added at −78 °C while the solution turns nearly colourless. After 15 min methyllithium (1.5 M in diethyl ether, 0.22 mL, 0.336 mmol, 1.1 equiv) was added again at −78 °C and the reaction mixture was warmed to −30 °C for 90 min. HCl was added (1 M in diethyl ether, 0.67 mL, 0.670 mmol, 2.2 equiv) and the reaction was warmed to rt for 2 h. The reaction was quenched with excess of HCOOH and the crude product was adsorbed directly on silica. Column chromatography (5% EtOAc, 0.1% HCOOH → 6% EtOAc, 0.15% HCOOH in pentane) gave 11 mg of *trans*-fatty acid with still 10% of the corresponding *cis*-isomer. The crude product was suspended in 80 mL of degassed *n*-hexane and treated with 6 µL of saturated solution of I_2_ in *n*-hexane. The reaction mixture was heated to 70 °C for 2.5 h. After removal of solvent fatty acid **3**, a pale yellow solid, was obtained as *trans*-isomer without loss of material. It should be mentioned that the compound is first dissolved in tiny amounts of THF before hexane is added to yield a fine suspension. ^1^H NMR (300 MHz, THF-*d*_8_) δ 1.20–1.65 (m, 16H), 2.20 (t, *J* = 7.3, 2H, C*H*_2_COOH), 2.34 (m, 2H), 6.92 (m, 1H), 7.17 (m, 1H), 7.55 (m, 2H), 7.83 (dd, *J* = 7.3, 2.6 Hz, 1H): ^13^C NMR (75 MHz, THF-*d*_8_) δ 29.58, 29.69, 29.75, 29.79, 29.94, 29.99, 33.72, 34.12, 119.7, 126.0, 126.5, 129.9, 131.5, 137.1, 153.7, 156.1, 173.9); IR (ATR) 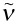 (cm^−1^): 2919, 2850, 1696, 1532, 1466, 1428, 1302, 1263, 1237, 1209, 967, 907, 756; HRMS (ESI) *m*/*z*: calcd for C_19_H_26_N_2_O_2_SNa^+^, 369.16072; found, 369.16088.

**(*E*)-3-(Benzo[*c*][1,2,5]thiadiazol-4-yl)prop-2-en-1-ol (6):** To a solution of triethyl phosphonoacetate (266 mg, 1.18 mmol, 1.3 equiv) in THF (5 mL) was added *n*-butyllithium (2.5 M, 0.48 mL, 1.18 mmol, 1.3 equiv) dropwise at 0 °C. After 15 min aldehyde **1** was added as solid. Stirring was continued for 1 h at 0 °C. At −78 °C BF_3_·OEt_2_ (0.15 mL, 1.18 mmol, 1.3 equiv) was added and directly afterwards DIBAL (1 M in hexane, 3.7 mL, 3.65 mmol, 4 equiv) was added. Stirring was continued at −78 °C for 1 h. The reaction was quenched with excess of MeOH at −78 °C, warmed to rt and treated with excess of Rochelle salt solution. Stirring continued vigorously at rt for 1 h. After an aqueous work-up with EtOAc the crude product was submitted to column chromatography on silica gel (30% EtOAc in pentane) to give 153 mg (0.80 mmol, 87%) of pure alcohol **6**, a pale yellow solid, as *trans*-isomer. ^1^H NMR (300 MHz, CDCl_3_) δ 1.95 (s, 1H, O*H*), 4.48 (dd, *J* = 5.3, 1.4 Hz, 2H, C*H*_2_OH), 7.11 (m, 1H), 7.26 (m, 1H), 7.54 (m, 2H), 7.88 (dd, *J* = 7.8, 2.0 Hz, 1H); ^13^C NMR (75 MHz, CDCl_3_) δ 63.8, 120.3, 126.3, 126.8, 129.5, 130.0, 134.4, 153.1, 155.4; IR (ATR) 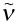 (cm^−1^): 3315, 3067, 2898, 2854, 1533, 1084, 1044, 996, 969, 908, 754; HRMS (EI) *m*/*z*: calcd for C_9_H_8_N_2_OS, 192.03573; found, 192.03451.

**(10*E*,12*E*)-13-(Benzo[*c*][1,2,5]thiadiazol-4-yl)tridec-10,12-dienoic acid (7):** To a solution of alcohol **6** (160 mg, 0.832 mmol, 1.0 equiv) in DCM (8 mL) was added MnO_2_ (1.5 g, 16.6 mmol, 20 equiv) at rt. Stirring was continued for 45 min. A mixture of SiO_2_/Al_2_O_3_ (1:1) was added and the solvent removed to obtain the adsorbed crude product. A short column filtration (20% EtOAc in pentane) gave 102 mg of the corresponding aldehyde which was used directly in the next step. The corresponding Wittig salt (363 mg, 0.707 mmol, 1.3 equiv) was suspended in THF (5 mL). NaHMDS (1 M in THF, 1.40 mL, 1.40 mmol, 2.6 equiv) was added at rt. Stirring was continued until a red solution was established (10 min). At 0 °C freshly prepared aldehyde dissolved in THF (2 mL) was added and stirring continued for 1 h at 0 °C. The reaction was quenched with some excess of HCOOH and the reaction mixture was directly adsorbed on silica. Column chromatography (5% EtOAc, 0.1% HCOOH → 7% EtOAc, 0.1% HCOOH in pentane) gave 100 mg (0.290 mmol, 54%) of fluorescent fatty acid **7** as *cis*–*trans* mixture (≈15% *cis*-isomer). Isomerization in 300 mL of degassed *n*-hexane with 30 µL of saturated I_2_ solution in *n*-hexane at 65 °C for 90 min gave compound **7**, a yellow solid, as pure all-*trans*-isomer after removal of solvents. It should be mentioned that the compound is first dissolved in tiny amounts of THF before hexane is added to yield a fine suspension. ^1^H NMR (300 MHz, THF-*d*_8_) δ 1.20–1.65 (m, 12H), 2.13–2.25 (m, 4H, C*H*_2_COOH, -C=CC*H_2_*), 5.99 (dd, *J* = 14.7, 7.1 Hz, 1H), 6.34 (m, 1H), 6.96 (d, *J* = 15.7 Hz, 1H), 7.50–7.63 (m, 2H), 7.72 (dd, *J* = 15.7, 10.6 Hz, 1H), 7.78–7.88 (m, 1H); ^13^C NMR (75 MHz, THF-*d*_8_) δ 30.11, 30.13, 30.23, 30.28, 30.33, 33.84, 34.25, 120.3, 126.9, 126.9, 130.5, 132.1, 132.4, 135.8, 138.3, 154.1, 156.7, 174.5; IR (ATR) 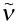 (cm^−1^): 3031, 2994, 2919, 2851, 1703, 1469, 1415, 1310, 1287, 1257, 1219, 988, 897, 829, 747; HRMS (ESI) *m*/*z*: calcd for C_19_H_24_N_2_O_2_SNa^+^, 367.14507; found, 369.14518.

**Methyl (2*E*,4*E*)-5-(benzo[*c*][1,2,5]thiadiazol-4-yl)penta-2,4-dienoate (9):** To a solution of conjugated phosphonate **8** (189 mg, 0.792 mmol, 1.3 equiv) in THF (5 mL) was added *n*-butyllithium (2.5 M, 0.32 mL, 0.792 mmol, 1.3 equiv) at 0 °C. Stirring was continued at this temperature for 15 min. A solution of aldehyde **1** (100 mg, 0.609 mmol, 1 equiv) in THF (2 mL) was added dropwise at 0 °C and strirring continued for 1 h. The reaction was then quenched with aqueous saturated NH_4_Cl solution and worked-up with DCM. Column chromatography on silica gel (5% EtOAc, 20% DCM in pentane) gave 124 mg (0.503 mmol, 83%) of highly fluorescent ester **9** as a yellow solid. ^1^H NMR (300 MHz, CDCl_3_) δ 3.80 (s, 3H, OMe), 6.16 (m, 1H), 7.31 (m, 1H), 7.45–7.65 (m, 3H), 7.80–8.00 (m, 2H); ^13^C NMR (75 MHz, CDCl_3_) δ 51.65, 121.7, 122.4, 128.9, 129.4, 129.5, 131.7, 136.0, 145.0, 152.9, 155.5, 167.4; IR (ATR) 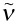 (cm^−1^): 2993, 2956, 1711, 1619, 1528, 1462, 1330, 1244, 1225, 1137, 990, 837, 750; HRMS (ESI) *m*/*z*: calcd for C_12_H_10_N_2_O_2_SNa^+^, 269.03552; found, 269.03557.

**(2*E*,4*E*)-5-(Benzo[*c*][1,2,5]thiadiazol-4-yl)penta-2,4-dien-1-ol (10):** To a solution of ester **9** (124 mg, 0.503 mmol, 1.0 equiv) in DCM (3 mL) was added DIBAL (1 M in hexane, 1.51 mL, 1.51 mmol, 3 equiv) at −20 °C. Stirring continued for 1 h at −20 °C and for 1 h at 0 °C. MeOH was added, then Rochelle salt solution and vigorous stirring continued for 1 h at rt. After an aqueous work-up the crude product was submitted to column chromatography on silica gel (20% EtOAc, 30% DCM → 30% EtOAc, 30% DCM in pentane) to give 85 mg (0.390 mmol, 77%) of the pure alcohol **10** as a yellow solid. ^1^H NMR (300 MHz, CDCl_3_) δ 1.75 (s, 1H, OH), 4.31 (d, *J* = 5.5 Hz, 2H, C*H*_2_OH), 6.14 (m, 1H), 6.54 (m, 1H), 7.03 (d, *J* = 15.7 Hz, 1H), 7.48–7.58 (m, 2H) 7.67 (dd, *J* = 15.7, 10.4 Hz, 1H), 7.85 (dd, *J* = 7.8, 2.0 Hz, 1H); ^13^C NMR (75 MHz, CDCl_3_) δ 63.34, 120.1, 126.6, 128.4, 129.6, 130.6, 131.7, 133.5, 134.6, 153.1, 155.5); IR (ATR) 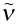 (cm^−1^): 3416, 3358, 3014, 2991, 1529, 1483, 1275, 1155, 1094, 1066, 980, 905, 837, 742; HRMS (ESI) *m*/*z*: calcd for C_11_H_10_N_2_OSNa^+^, 241.04060; found, 241.04071.

**(8*E*,10*E*,12*E*)-8-(Benzo[*c*][1,2,5]thiadiazol-4-yl)tridec-8,10,12-trienoic acid (11c):** To a solution of **10** (40 mg, 0.183 mmol, 1.0 equiv) in DCM (1.5 mL) was added MnO_2_ (318 mg, 3.66 mmol, 20 equiv) at rt. Stirring was continued for 45 min. A mixture of SiO_2_/Al_2_O_3_ (1:1) was added and the solvent removed to obtain the adsorbed crude product. A short column filtration (20% EtOAc in pentane) gave 34 mg of the corresponding aldehyde (86%) which was used directly in the next step. The corresponding Wittig salt (116 mg, 0.238 mmol, 1.3 equiv) was suspended in THF (2 mL). NaHMDS (1 M in THF, 0.78 mL, 0.480 mmol, 2.6 equiv) was added at rt. Stirring was continued until a red solution was established (10 min). At 0 °C freshly prepared aldehyde dissolved in THF (1 mL) was added and stirring continued for 1 h at 0 °C. The reaction was quenched with HCOOH (3 equiv) and directly adsorbed on silica/Al_2_O_3_ (1:1). Column chromatography (10% EtOAc in pentane to remove rests of aldehyde, then 5% EtOAc, 0.1% HCOOH → 6% EtOAc, 0.15% HCOOH in pentane) gave 20 mg (0.058 mmol, 37%) of fatty acid. In some cases size exclusion chromatography in CHCl_3_ was helpful to remove rests of Wittig salt. Isomerization was performed in *n*-hexane (200 mL, degassed) with 15 µL of saturated I_2_-solution in *n*-hexane for 90 min at 65 °C. It should be mentioned that the compound was first dissolved in tiny amounts of THF before hexane was added to yield a fine suspension. After removal of solvents fatty acid **11c**, a yellow solid, was obtained as pure all-*trans* isomer. ^1^H NMR (300 MHz, THF-*d*_8_) δ 1.20–1.65 (m, 8H), 2.06–2.26 (m, 4H, C*H*_2_COOH, -C=CC*H_2_*), 5.83 (dd, *J* = 15.1, 7.1 Hz, 1H), 6.21 (m, 1H), 6.45 (m, 2H), 7.03 (d, *J* = 15.6 Hz, 1H), 7.50–7.66 (m, 2H), 7.72–7.86 (m, 2H); ^13^C NMR (75 MHz, THF-*d*_8_) δ 25.78, 29.88, 29.94, 30.11, 33.75, 34.21, 120.4, 127.1, 128.2, 130.5, 131.8, 132.0, 132.4, 135.7, 136.5, 137.2, 154.1, 156.7, 174.4; IR (ATR) 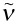 (cm^−1^): 3009, 2927, 2853, 1695, 1526, 1265, 993, 906, 750; HRMS (ESI) *m*/*z*: calcd for C_19_H_24_N_2_O_2_SNa^+^, 365.12942; found, 365.12956.

**(16*E*,18*E*,20*E*)-21-(Benzo[*c*][1,2,5]thiadiazol-4-yl)henicos-16,18,20-trienoic acid (11d):** To a solution of alcohol **10** (40 mg, 0.183 mmol, 1.0 equiv) in DCM (1.5 mL) was added MnO_2_ (318 mg, 3.66 mmol, 20 equiv) at rt. Stirring was continued for 45 min. A mixture of SiO_2_/Al_2_O_3_ (1:1) was added and the solvent was removed to obtain the adsorbed crude product. A short column filtration (20% EtOAc in pentane) gave 31 mg of the corresponding aldehyde (78%) which was used directly in the next step. The corresponding Wittig salt (111 mg, 0.186 mmol, 1.3 equiv) was suspended in THF (1.5 mL). NaHMDS (1 M in THF, 0.37 mL, 0.372 mmol, 2.6 equiv) was added at rt. Stirring was continued until a red solution was established (10 min). At 0 °C freshly prepared aldehyde dissolved in THF (1 mL) was added and stirring continued for 1 h at 0 °C. The reaction was quenched with HCOOH (3 equiv) and directly adsorbed on silica/Al_2_O_3_ (1:1). Column chromatography (10% EtOAc in pentane to remove rests of aldehyde, then 5% EtOAc, 0.1% HCOOH → 7% EtOAc, 0.1% HCOOH in pentane) gave 45 mg (0.099 mmol, 54%) of fatty acid. Isomerization was performed in *n*-hexane (300 mL, degassed) with 20 µL of saturated I_2_-solution in *n*-hexane for 90 min at 65 °C. It should be mentioned that the compound was first dissolved in tiny amounts of THF before hexane was added to yield a fine suspension. After removal of solvents fatty acid **11d**, a yellow solid, is usually obtained as pure all-*trans*-isomer. ^1^H NMR (300 MHz, THF-*d*_8_) δ 1.20–1.65 (m, 8H), 2.06–2.26 (m, 4H, C*H*_2_COOH, -C=CC*H_2_*), 5.82 (dd, *J* = 15.1, 7.1 Hz, 1H), 6.20 (m, 1H), 6.45 (m, 2H), 7.03 (d, *J* = 15.6 Hz, 1H), 7.50–7.66 (m, 2H), 7.72–7.86 (m, 2H); ^13^C NMR (75 MHz, THF-*d*_8_) δ 29.59, 29.65, 29.74, 29.80, 29.93, 29.95, 30.05, 30.08, 33.28, 33.72, 119.8, 126.6, 127.7, 123.0, 131.3, 131.5, 131.9, 135.2, 135.9, 136.8, 153.6, 156.2, 173.9; IR (ATR) 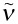 (cm^−1^): 3010, 2920, 2849, 1696, 1527, 1464, 1260, 993, 927, 802, 750, 725; HRMS (ESI) *m*/*z*: calcd for C_27_H_38_N_2_O_2_SNa^+^, 477.25462; found, 477.25542.

## Supporting Information

File 1 Copies of ^1^H and ^13^C NMR spectra.
