# Supplementary material for: Benzothiadiazole oligoene fatty acids: fluorescent dyes with large Stokes shifts
Source: Beilstein J Org Chem. 2016 Dec 14;12:2739–47. doi: 10.3762/bjoc.12.270 (PMC5238556; doi:10.3762/bjoc.12.270)
Supplement: File 1 — Copies of 1H and 13C NMR spectra. [file Beilstein_J_Org_Chem-12-2739-s001.pdf]

Supporting Information

for

Benzothiadiazole oligoene fatty acids: fluorescent dyes with large Stokes shifts

Lukas J. Patalag and Daniel B. Werz*

Address: Institut für Organische Chemie, Technische Universität Braunschweig, Hagenring 30, 38106 Braunschweig, Germany

Email: Prof. Dr. Daniel B. Werz* - d.werz@tu-braunschweig.de

* Corresponding author

Copies of ^{1}H and ^{13}C NMR spectra

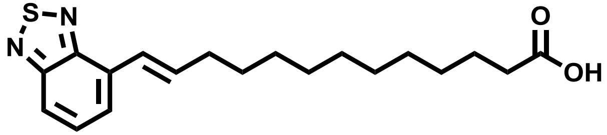

3

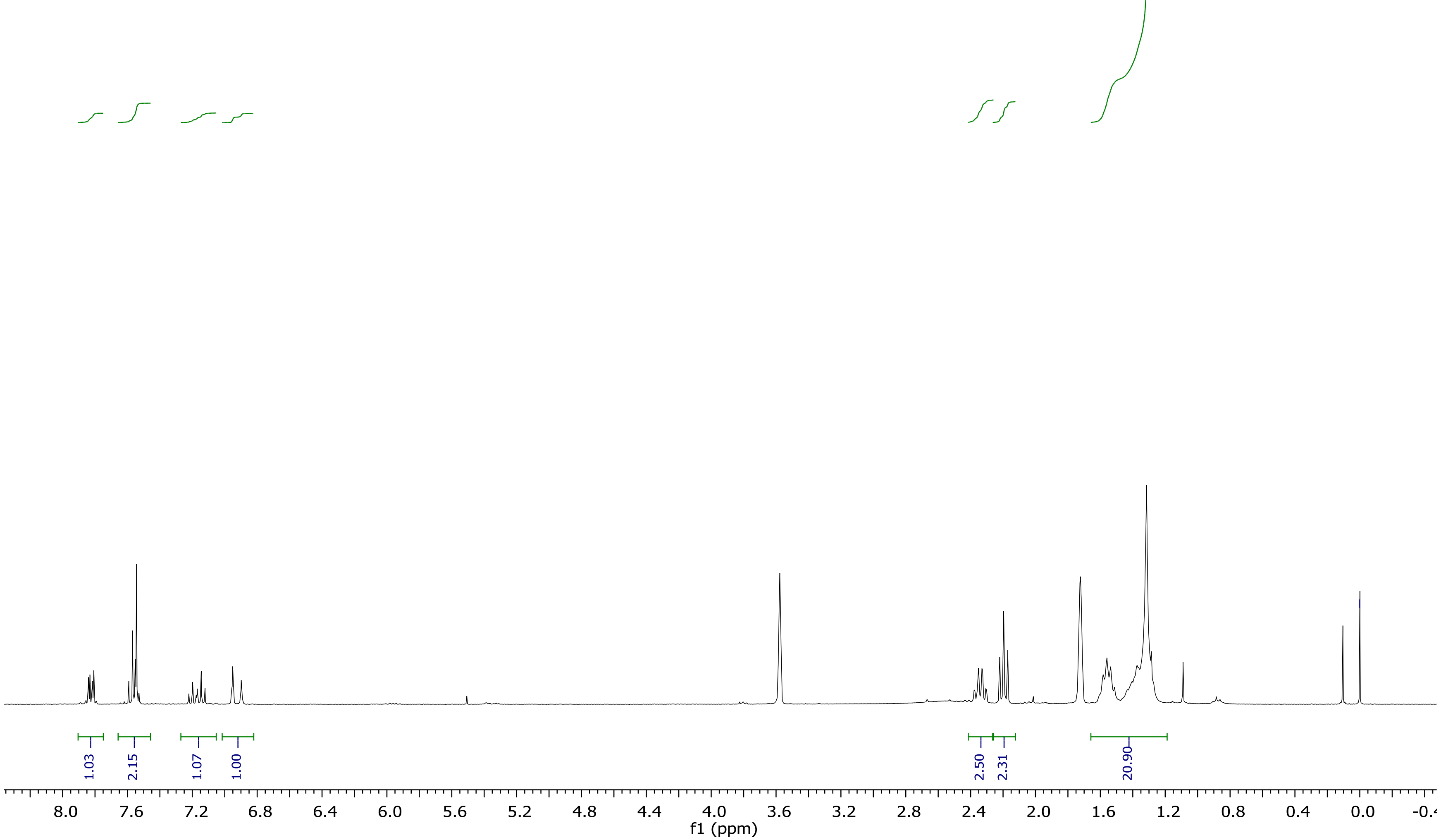
0.00
1.03
2.15
1.07
1.00
2.50
2.31
20.90
8.0
7.6
7.2
6.8
6.4
6.0
5.6
5.2
4.8
4.4
4.0
3.6
3.2
2.8
2.4
2.0
1.6
1.2
0.8
0.4
0.0
f1 (ppm)

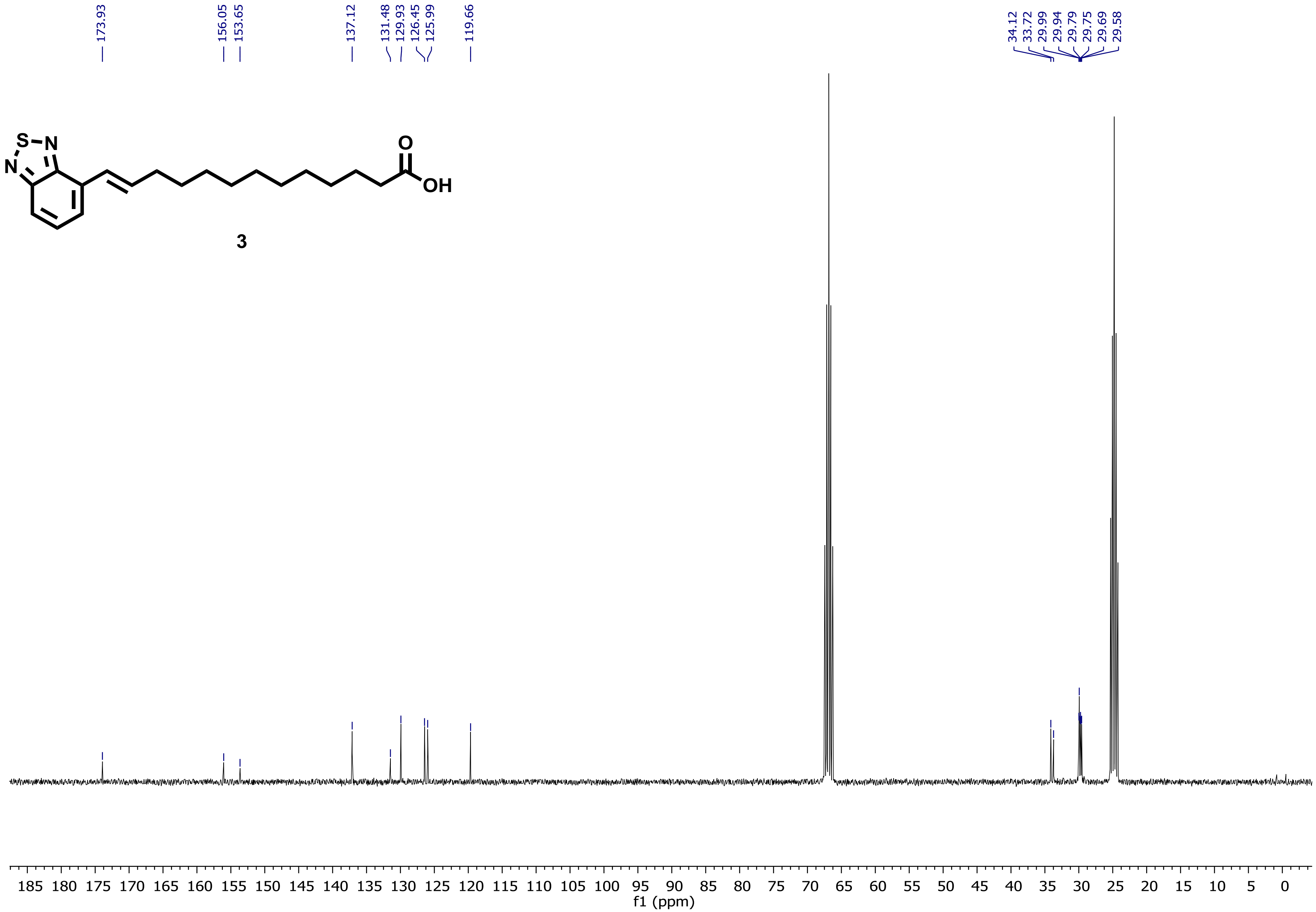

173.93
156.05
153.65
137.12
131.48
129.93
126.45
125.99
119.66
34.12
33.72
29.99
29.94
29.79
29.75
29.69
29.58
S
N
N
O
OH
3
185 180 175 170 165 160 155 150 145 140 135 130 125 120 115 110 105 100 95 90 85 80 75 70 65 60 55 50 45 40 35 30 25 20 15 10 5 0
f1 (ppm)

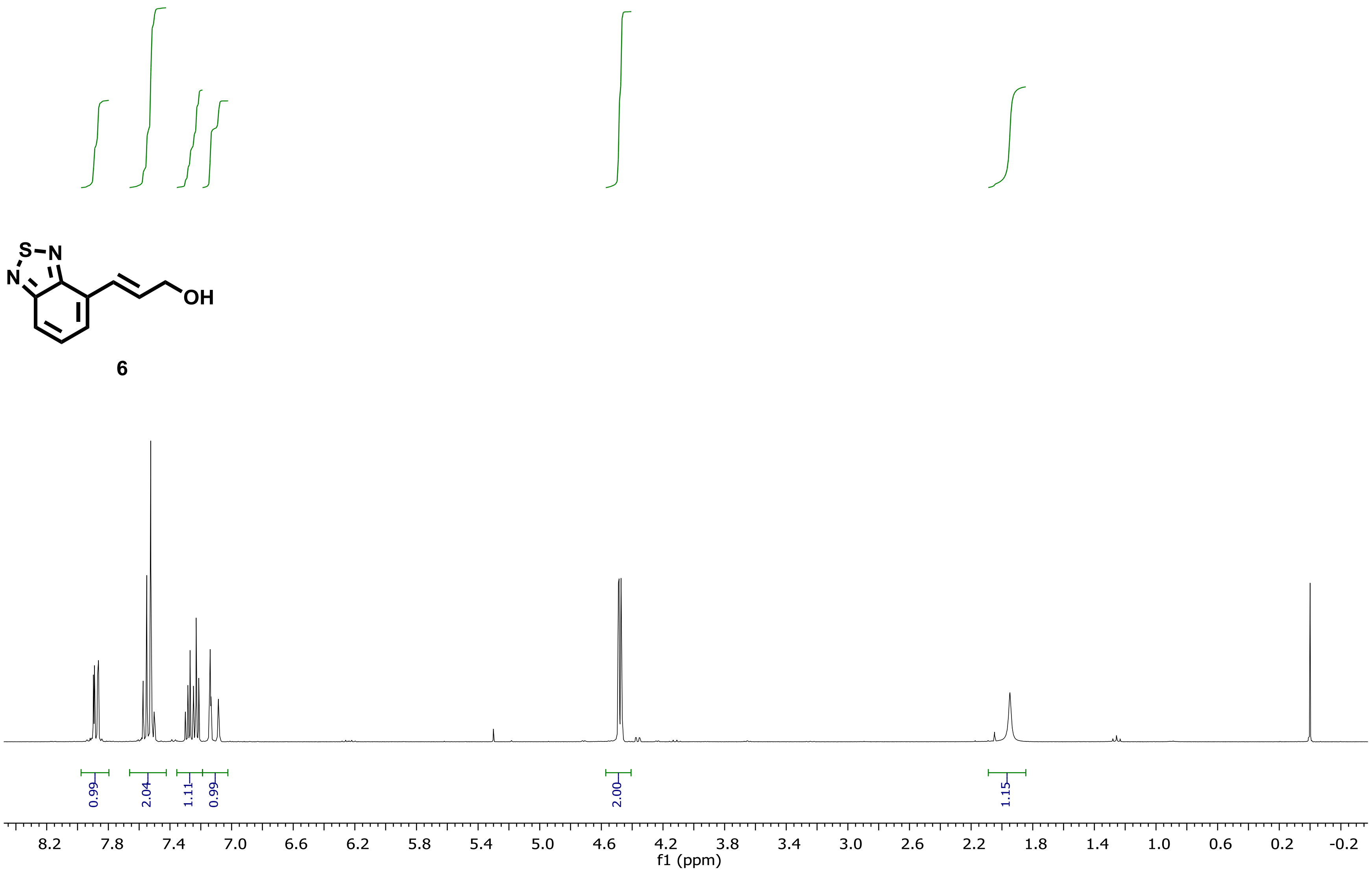
S
N
N
OH
0.99
2.04
1.11
0.99
2.00
1.15
8.2
7.8
7.4
7.0
6.6
6.2
5.8
5.4
5.0
4.6
4.2
3.8
3.4
3.0
2.6
2.2
1.8
1.4
1.0
0.6
0.2
-0.2
f1 (ppm)

6

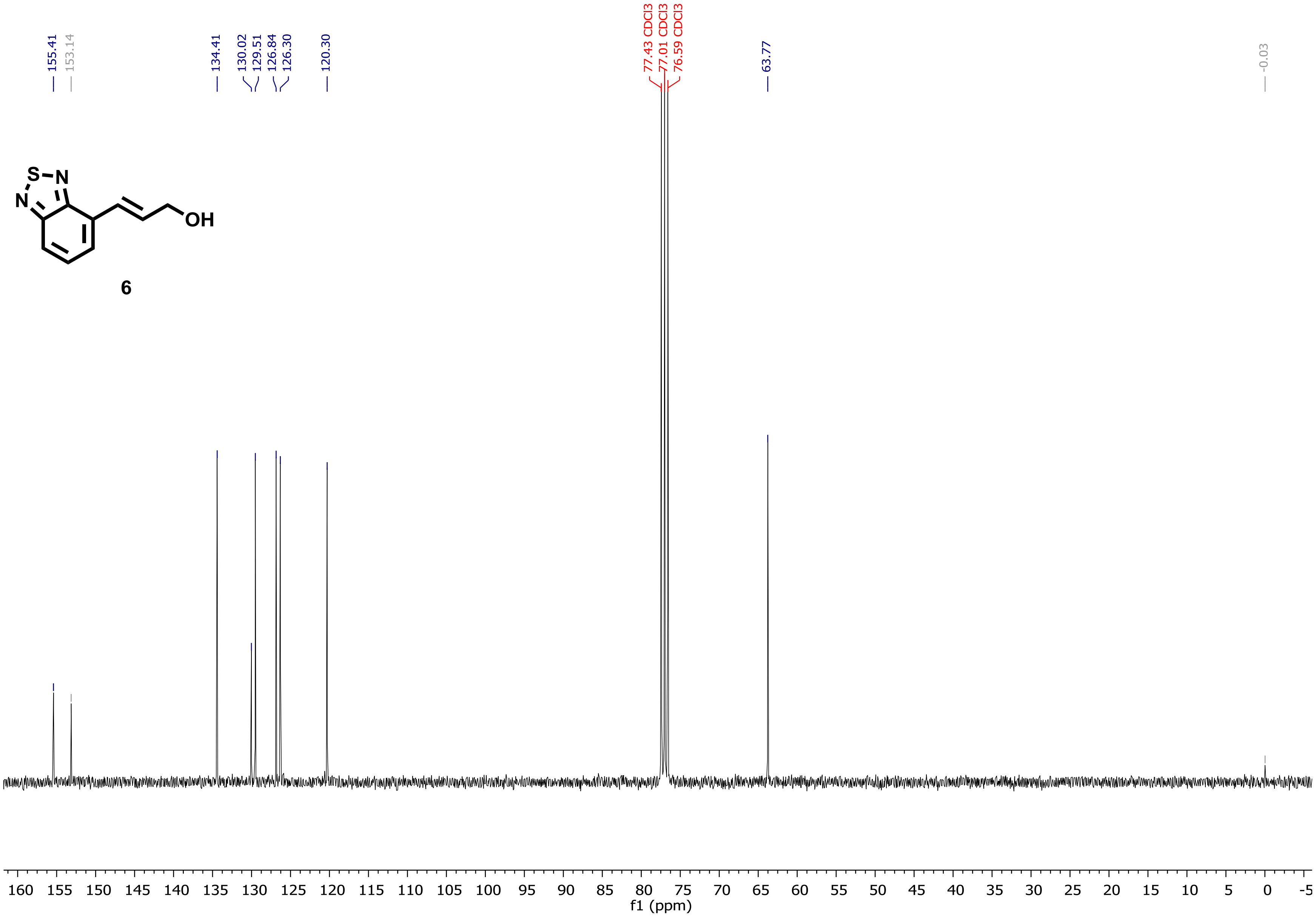
155.41
153.14
134.41
130.02
129.51
126.84
126.30
120.30
77.43 CDCl3
77.01 CDCl3
76.59 CDCl3
63.77
-0.03
S
N
N
OH
6
160 155 150 145 140 135 130 125 120 115 110 105 100 95 90 85 80 75 70 65 60 55 50 45 40 35 30 25 20 15 10 5 0 -5
f1 (ppm)

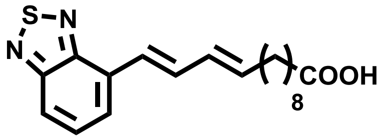
S
N
N
COOH
8

7

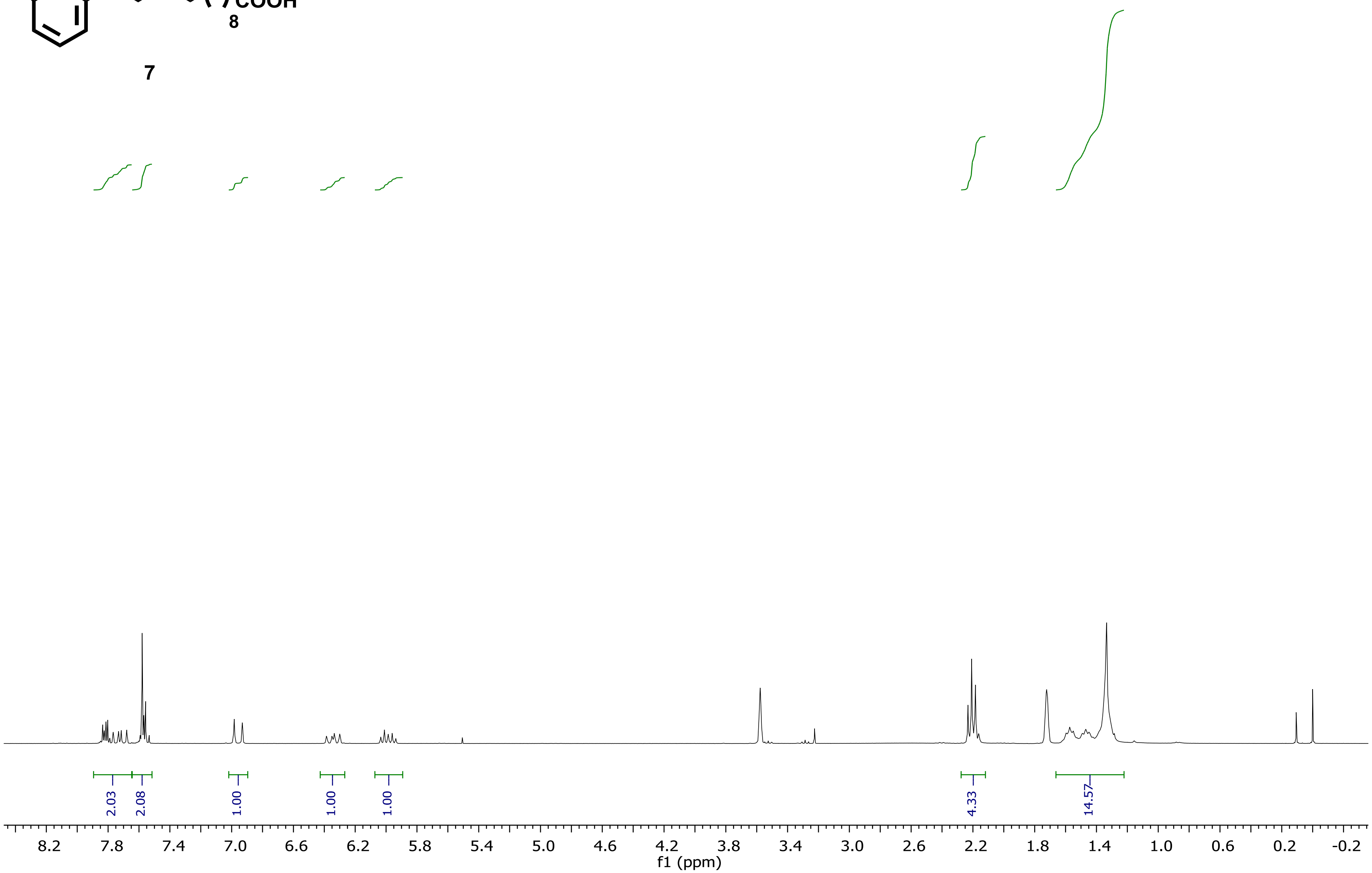
2.03
2.08
1.00
1.00
1.00
4.33
14.57
8.2
7.8
7.4
7.0
6.6
6.2
5.8
5.4
5.0
4.6
4.2
3.8
3.4
3.0
2.6
2.2
1.8
1.4
1.0
0.6
0.2
-0.2
f1 (ppm)

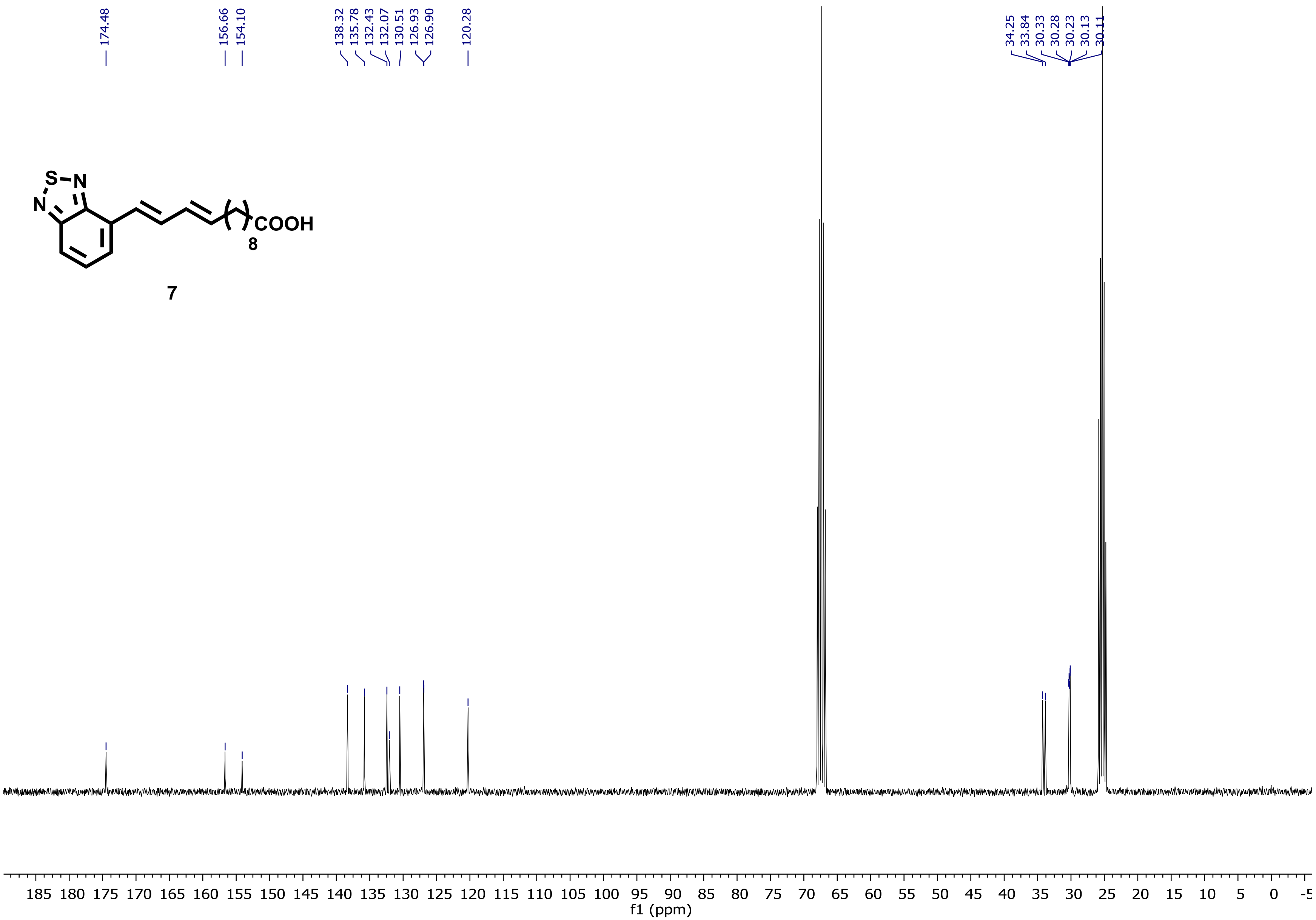

174.48
156.66
154.10
138.32
135.78
132.43
132.07
130.51
126.93
126.90
120.28
34.25
33.84
30.33
30.28
30.23
30.13
30.11
S
N
N
COOH
8
7
f1 (ppm)

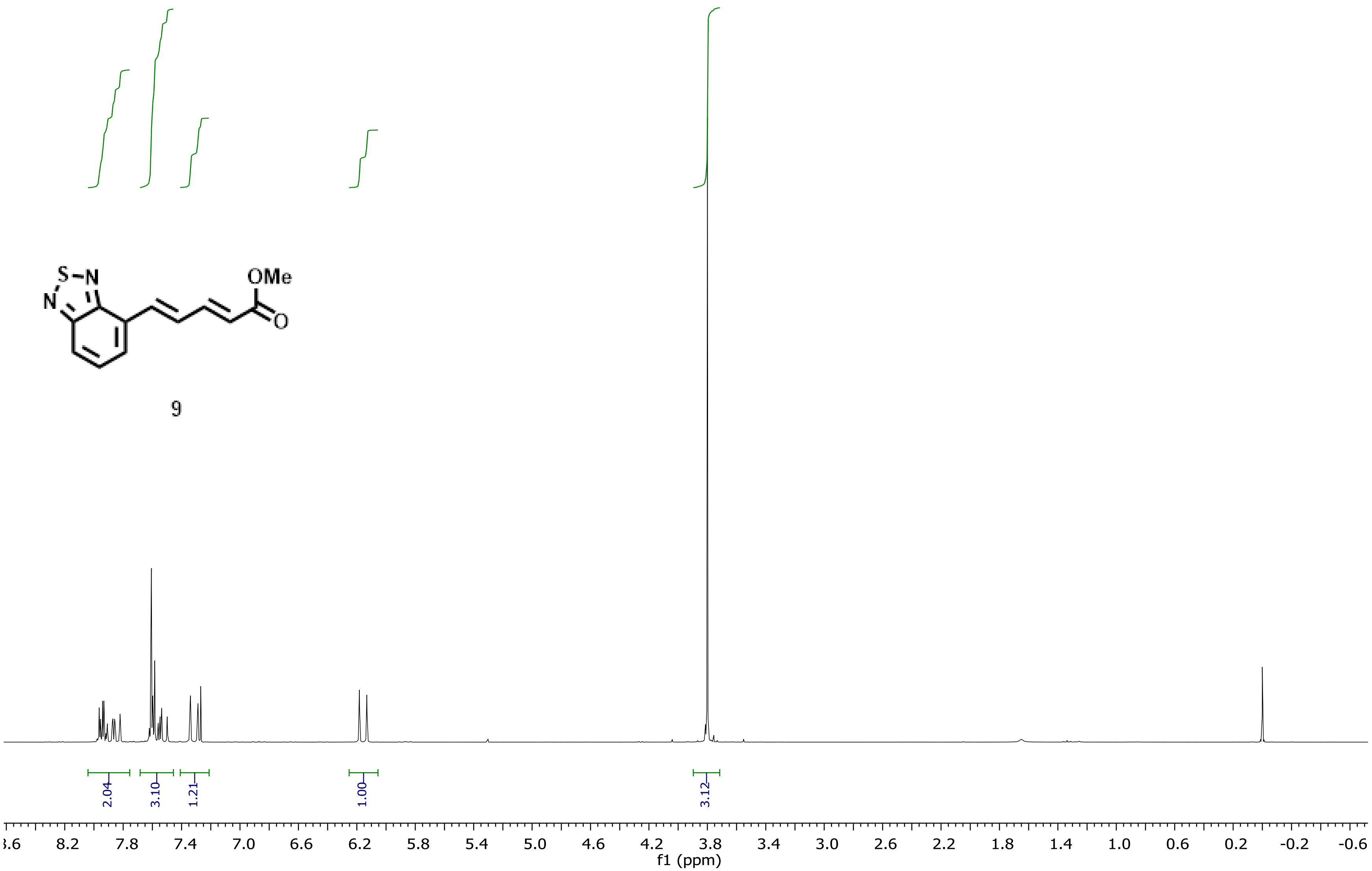

S
N
N
OMe
O
9
2.04
3.10
1.21
1.00
3.12
8.6 8.2 7.8 7.4 7.0 6.6 6.2 5.8 5.4 5.0 4.6 4.2 3.8 3.4 3.0 2.6 2.2 1.8 1.4 1.0 0.6 0.2 -0.2 -0.6
f1 (ppm)

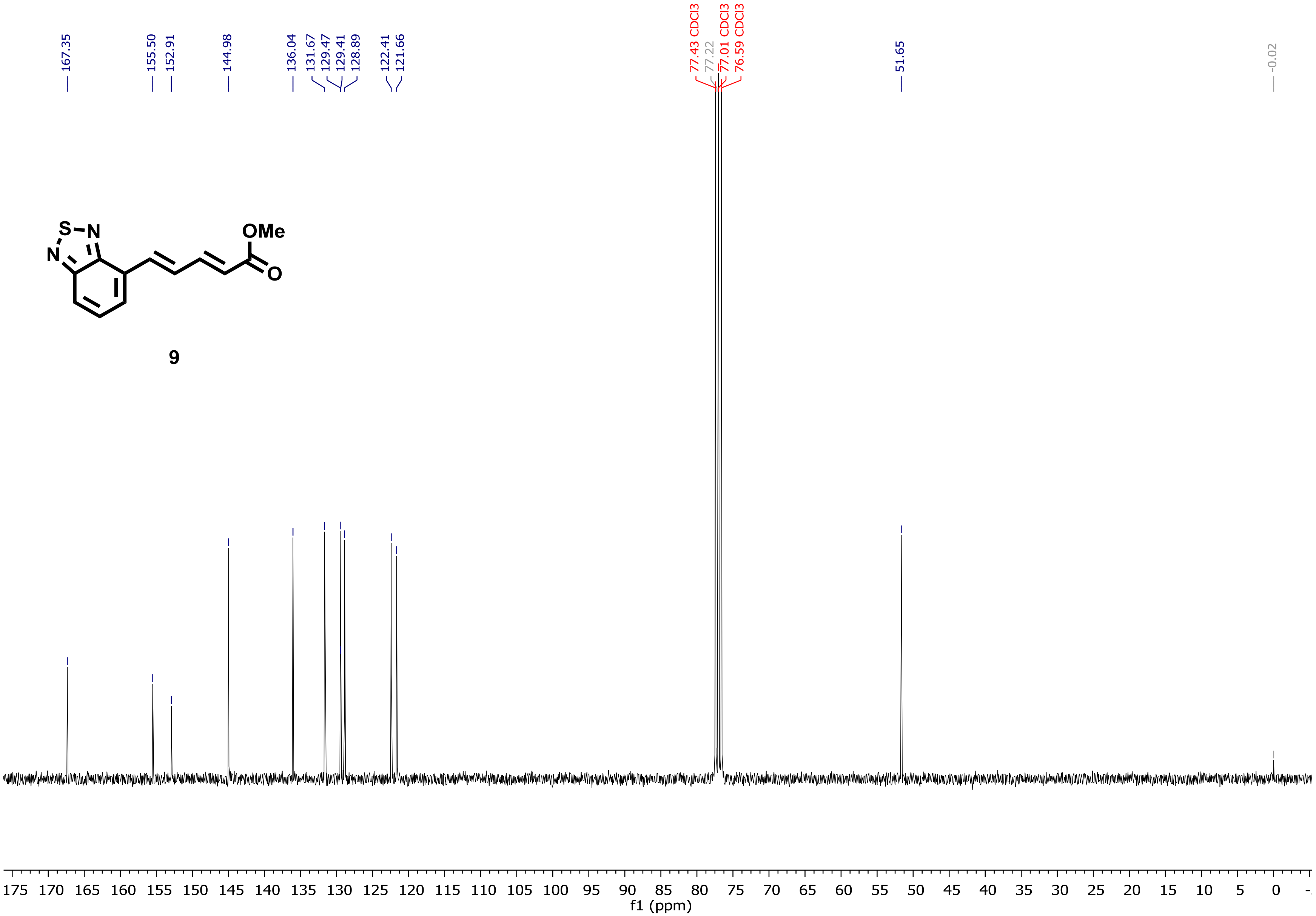

167.35
155.50
152.91
144.98
136.04
131.67
129.47
129.41
128.89
122.41
121.66
77.43 CDCl3
77.22
77.01 CDCl3
76.59 CDCl3
51.65
-0.02
S
N
N
OMe
O
175 170 165 160 155 150 145 140 135 130 125 120 115 110 105 100 95 90 85 80 75 70 65 60 55 50 45 40 35 30 25 20 15 10 5 0
f1 (ppm)

9

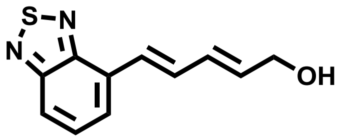
S
N
N
OH

10

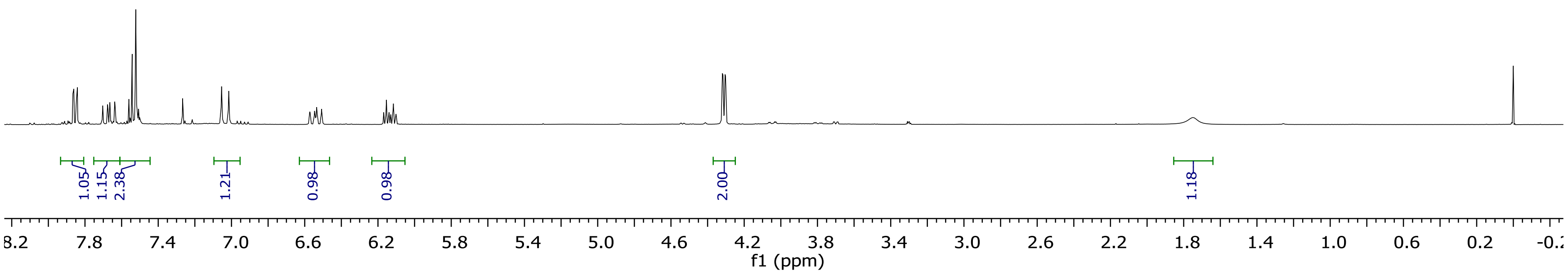
1.05
1.15
2.38
1.21
0.98
0.98
2.00
1.18
8.2
7.8
7.4
7.0
6.6
6.2
5.8
5.4
5.0
4.6
4.2
3.8
3.4
3.0
2.6
2.2
1.8
1.4
1.0
0.6
0.2
f1 (ppm)

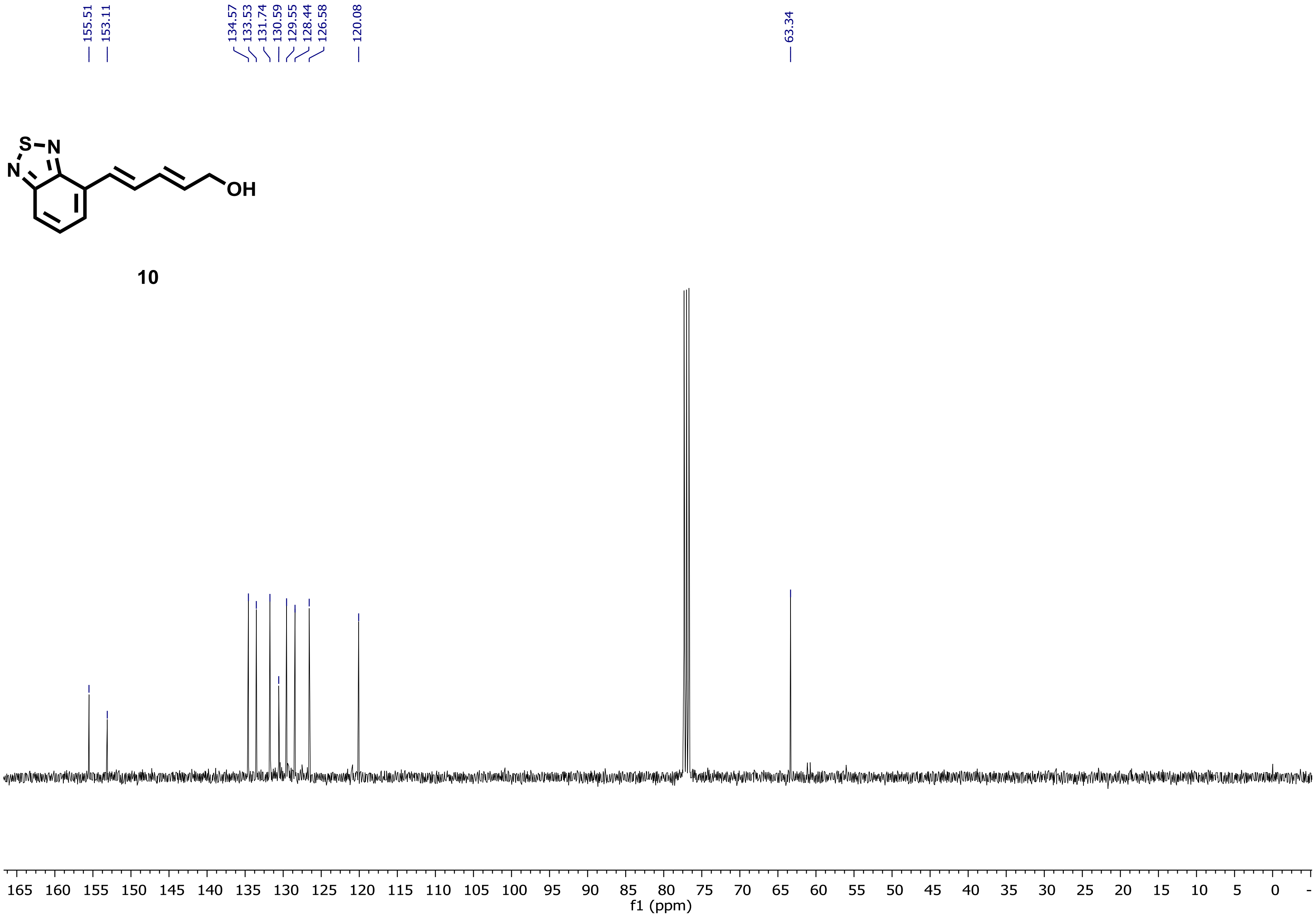
155.51
153.11
134.57
133.53
131.74
130.59
129.55
128.44
126.58
120.08
63.34
S
N
N
OH
10
165 160 155 150 145 140 135 130 125 120 115 110 105 100 95 90 85 80 75 70 65 60 55 50 45 40 35 30 25 20 15 10 5 0 -
f1 (ppm)

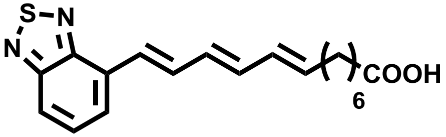
S–N
N
COOH
6

11c

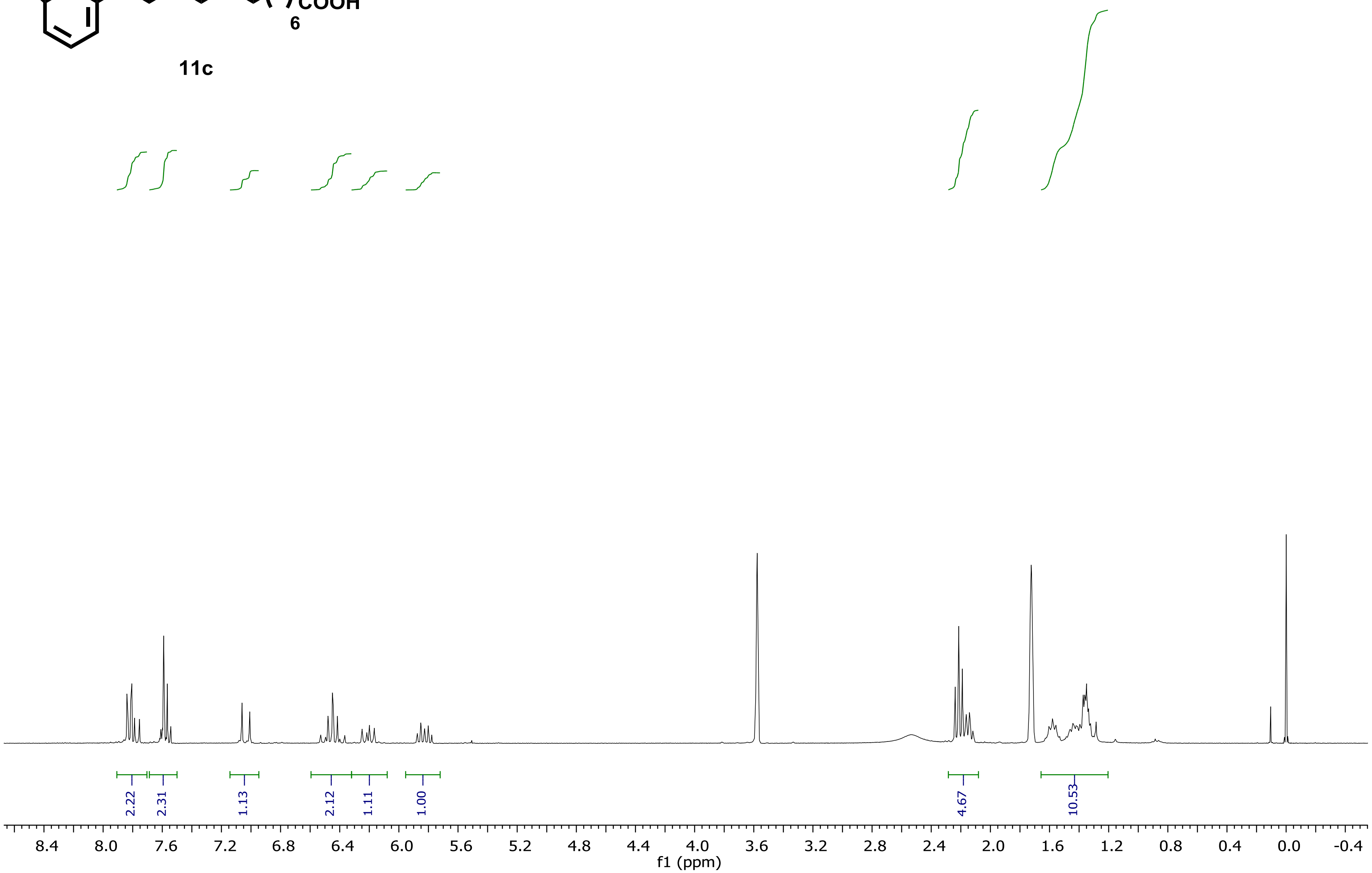
2.22
2.31
1.13
2.12
1.11
1.00
4.67
10.53
8.4 8.0 7.6 7.2 6.8 6.4 6.0 5.6 5.2 4.8 4.4 4.0 3.6 3.2 2.8 2.4 2.0 1.6 1.2 0.8 0.4 0.0 -0.4
f1 (ppm)

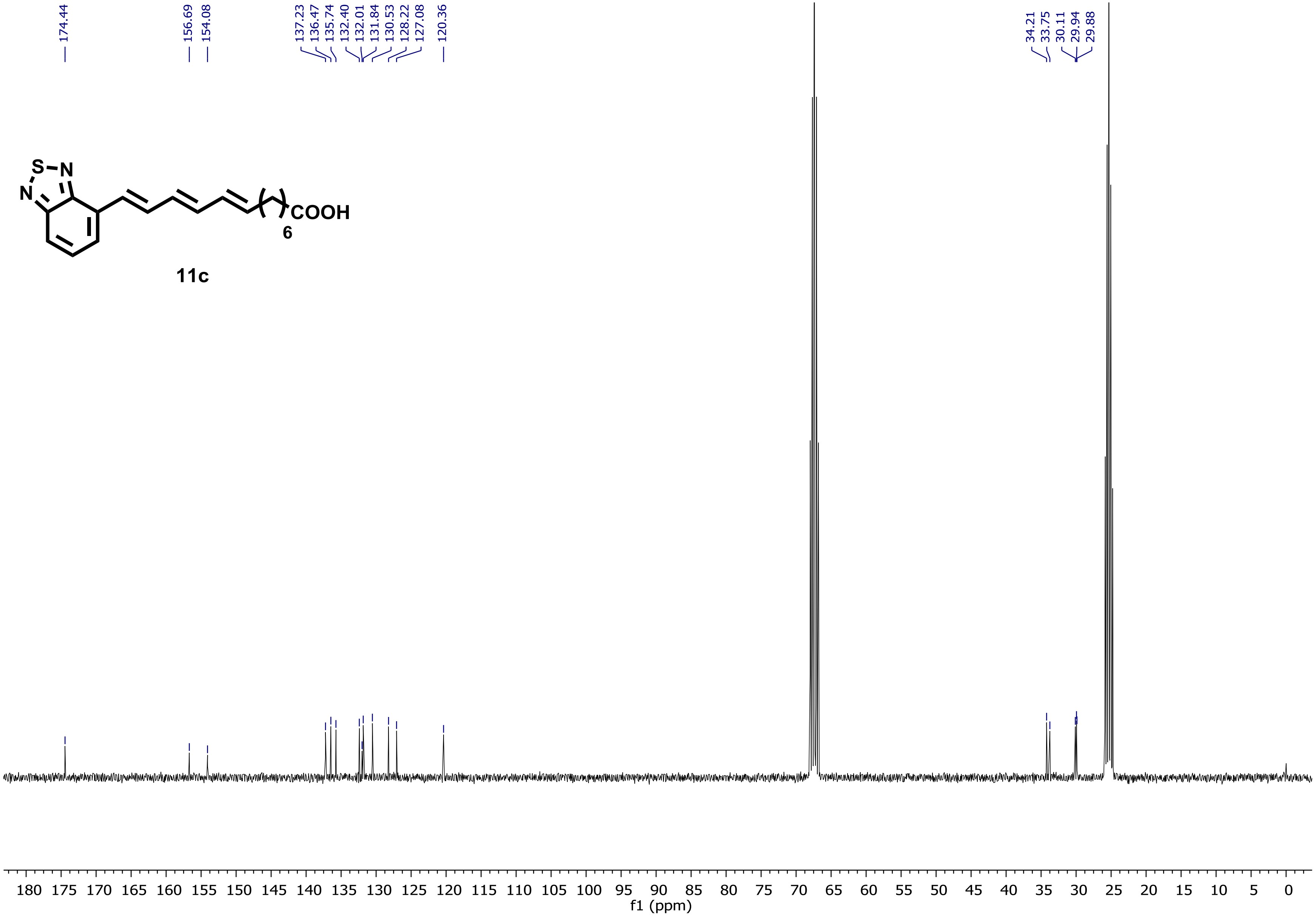
174.44
156.69
154.08
137.23
136.47
135.74
132.40
132.01
131.84
130.53
128.22
127.08
120.36
34.21
33.75
30.11
29.94
29.88
S
N
N
COOH
6
11c
180 175 170 165 160 155 150 145 140 135 130 125 120 115 110 105 100 95 90 85 80 75 70 65 60 55 50 45 40 35 30 25 20 15 10 5 0
f1 (ppm)

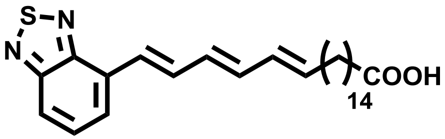

S
N
N
14
COOH

11d

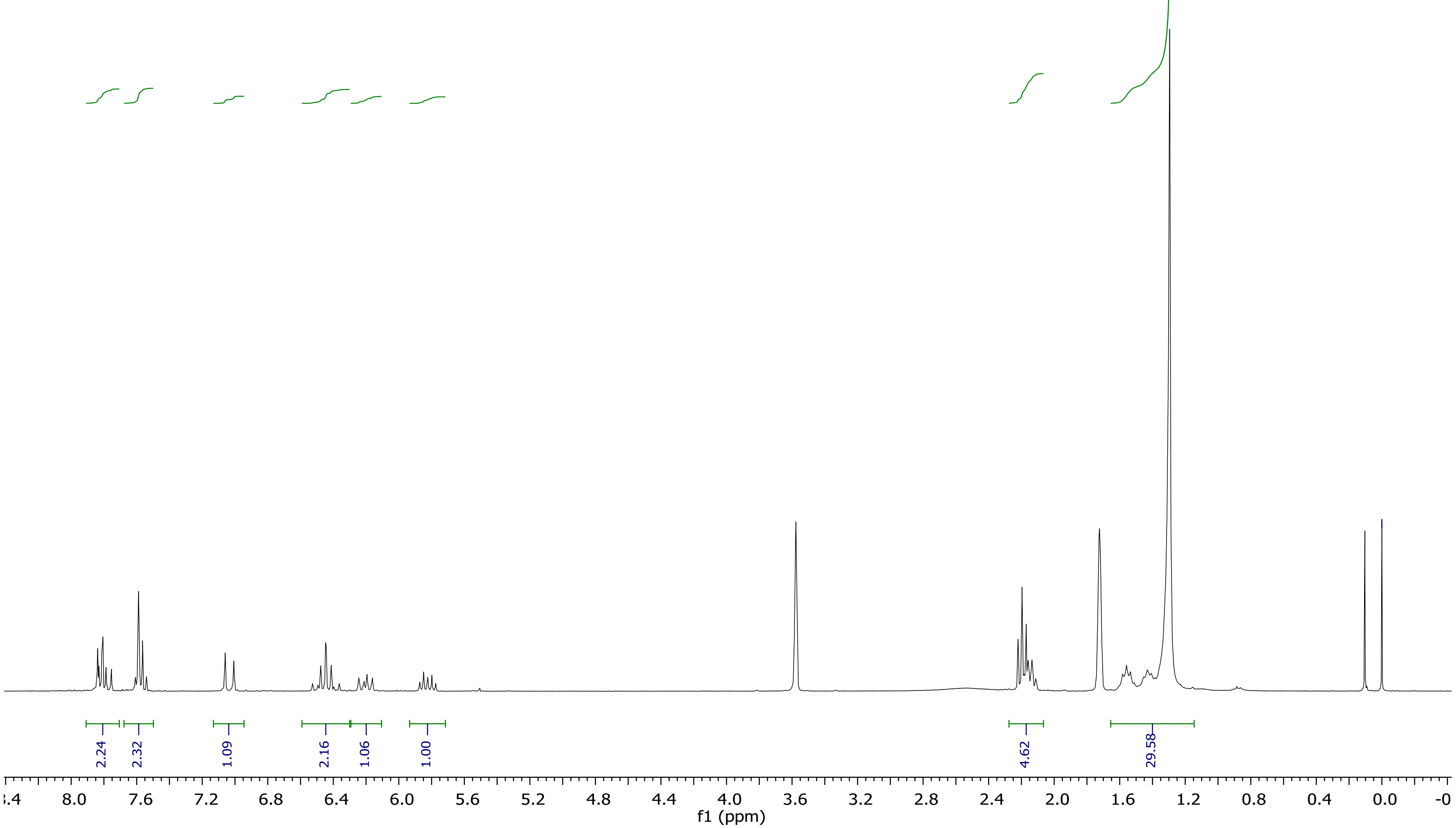

-0.00
2.24
2.32
1.09
2.16
1.06
1.00
4.62
29.58
8.0
7.6
7.2
6.8
6.4
6.0
5.6
5.2
4.8
4.4
4.0
3.6
3.2
2.8
2.4
2.0
1.6
1.2
0.8
0.4
0.0
f1 (ppm)

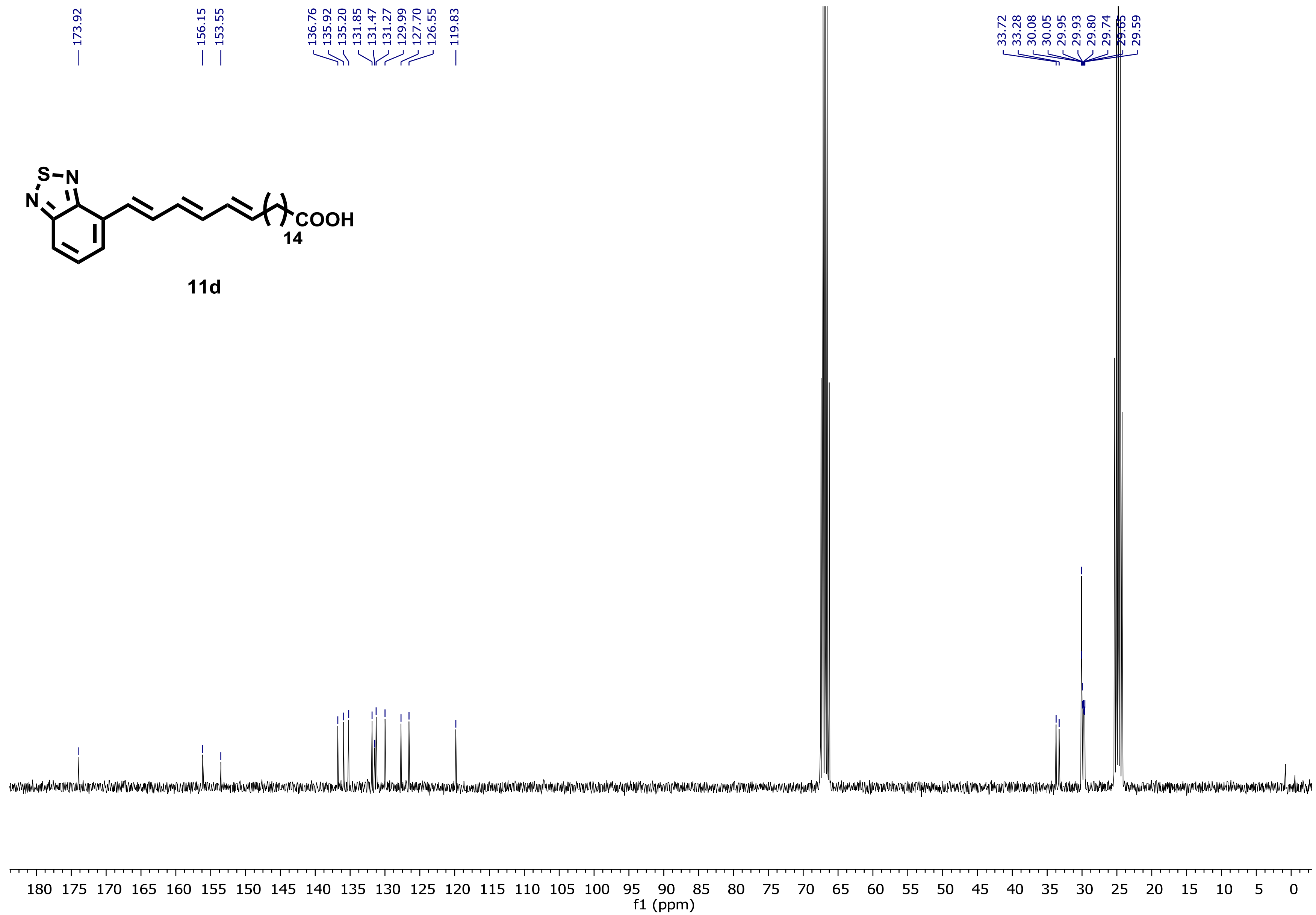
173.92
156.15
153.55
136.76
135.92
135.20
131.85
131.47
131.27
129.99
127.70
126.55
119.83
33.72
33.28
30.08
30.05
29.95
29.93
29.80
29.74
29.65
29.59
S
N
N
COOH
14
11d
180 175 170 165 160 155 150 145 140 135 130 125 120 115 110 105 100 95 90 85 80 75 70 65 60 55 50 45 40 35 30 25 20 15 10 5 0
f1 (ppm)
